# Non-leukodepleted red blood cell transfusion in sepsis patients: beyond oxygenation, is there a risk of inflammation?

**DOI:** 10.1186/s13054-014-0690-y

**Published:** 2014-12-08

**Authors:** Olivier Garraud, Adrien Chabert, Bruno Pozzetto, Fabrice Zeni, Fabrice Cognasse, Hind Hamzeh-Cognasse

**Affiliations:** Université de Lyon, GIMAP-EA3064, Saint Etienne, 42023 France; Institut National de Transfusion Sanguine (INTS), Paris, 75739 France; Département de Microbiologie Hôpital Nord, CHU de Saint-Etienne, Saint-Etienne, 42055 France; Service de Réanimation Médicale et Polyvalente Hôpital Nord, CHU de Saint-Etienne, Saint-Etienne, 42055 France; Etablissement Français du Sang Auvergne-Loire, Saint-Etienne, 42100 France

**Keywords:** ■■■

Donati and colleagues [1] evaluated the benefits of fresh, leukodepleted (LD) versus non-leukodepleted (nLD) erythrocyte transfusions on the microcirculation in sepsis patients. Oxygenation appeared equal in both groups. Differences between the two kinds of blood cells are presented in Table 1. In the reported study, there is a difference between the age of erythrocytes in the two groups (1.5 to 3 days for nLD versus 3.5 to 5 days for LD erythrocytes; a U test indicates *P* < 0.05); this suffices to influence oxygen delivery mediators (Figure 1). The content of leukocytes prior to (and after) leukodepletion were not tested, nor was the freeing - which is usually very fast - of secreted or docked, soluble biological response modifiers. Considering the dynamics of secreted biological response modifiers in platelet components [2], there must be differences within the two groups, which possibly influenced sepsis conditions. Residual plasma within erythrocytes given by female donors may include anti-human leukocyte antigen (HLA) antibodies which attack the recipient’s lung alveolar epithelium neutrophils, and as sepsis is characterized by the pathology of neutrophils that release microparticles and neutrophil extracellular traps that target lung epithelium [3], it may be feared here that the nLD condition aggravates pulmonary lesions.

**Table 1:** **Possible consequences of variation in transfusion conditions and the type of packed red blood cells administered**

**Variable**	**Primary or immediate consequences (efficacy)**	**Secondary or delayed consequences (hazards)**
**Total volume**	Possibly needs to be adjusted according to the patient's needs	Possibly needs to be adjusted to correct for anemia
**Hematocrit**	Possibly needs to be adjusted according to the patient's needs	Possibly needs to be adjusted to correct for anemia
**Residual plasma volume**	Possibly includes anti-HLA antibodies (from female donors)	Increases the risk of TRALI
		Increases the risk of inflammation and aggravates the risk of TRALI
	Possibly affects the amount of soluble, free biological response modifiers	
**Leukocytes**	No pre-test (possibly affects donor eligibility)	
	Pre-activation of leukocytes	Increases the risk of inflammation
	Release of biological response modifiers	
	Release of microparticles and neutrophil extracellular	Aggravates sepsis
	traps	Increases the risk of TRALI
	HLA antibody targets	Increases the risk of viral infections
	Infectious risk (intracellular viruses)	
**Age of blood**	Decreases the benefit of oxygen transport	
	Release of microparticles	Increases the risk of inflammation
	Expression of stress signals on red blood cells	
	Free iron release	Potentiates the risk of TRALI by stressing target
	NO and iNOS release	neutrophils
	Oxygenated lipid and lipid degradation	
		Possibly increases the risk of allo-immunization

HLA, human leukocyte antigen; iNOS, inducible nitric oxide synthase; NO, nitric oxide; TRALI, transfusion-related acute lung injury.

**Figure 1:**
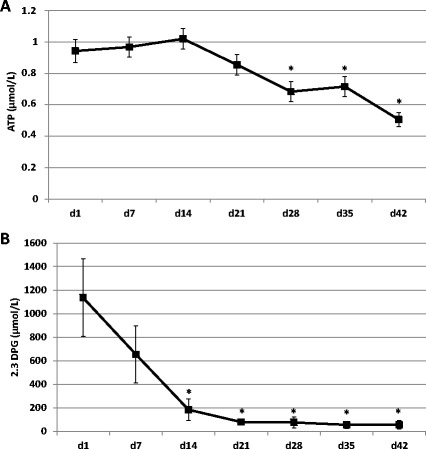
**ATP and 2,3-diphosphoglycerate (2.3-DPG) in packed red blood cells. (A)** ATP and **(B)** 2.3-DPG in packed red blood cells measured after 1, 7, 14, 21, 28, 35 and 42 days (d) of storage were re-evaluated by one-way analysis of variance. Inter-experiment differences in ATP and 2.3-DPG concentrations at different time points were analyzed by Wilcoxon paired test (XLSTAT® 2010 software, Addinsoft, Paris, France). *P*-values ≤0.05 were considered to be significant (*n = 10). PLT, platelet.

The re-evaluation of procedures is infrequent in transfusion despite the rapid evolution of techniques and materials; for example, differential stresses are inflicted on erythrocytes, depending on the collection process (aphaeresis versus conventional whole blood), with consequences for neutrophils and (vascular) endothelial cells upon transfusion [4]. Clinical investigations and registered trials such as Donati and colleagues are valuable.

## Authors' response

Abele Donati, Elisa Damiani, Erica Adrario, Rocco Romano, Paolo Pelaia and Can Ince

We thank Professor Garraud and colleagues for their interest in our study [1]. As underlined, the difference in the age of transfused red blood cells (RBCs) between the nLD group (4 (3.5 to 5) days) and the LD group (3 (1.5 to 3) days) may have influenced the RBC oxygen-delivery capacity. Stored RBCs lose their ability to release vasodilators (nitric oxide, ATP) during hypoxia [5,6]. We showed similar changes in microvascular reactivity (tissue oxygen saturation (StO_2_)-upslope) and oxygenation (StO_2_) after nLD or LD RBC transfusions [1]. This may indicate that oxygen-delivery mediators were not sufficiently affected to determine relevant variations in the response observed. Alternatively, heterogeneity in the study population prevented detection of subtle differences. Variability in the response to treatments is common during sepsis. The patient heterogeneity was underlined as a limitation of our investigation [1].

LD RBCs showed a more favorable effect on microcirculatory convective flow [1]. This may depend on the lower adhesiveness of LD RBCs to the endothelium [7]. The transfusion of nLD RBCs decreased blood flow velocity and increased glycocalyx damage markers [1]. Similar effects may reasonably occur in the lungs. As highlighted, anti-HLA antibodies in nLD blood from female donors may contribute to aggravate pulmonary lesions. Nonetheless, the evaluation of respiratory function went beyond our goals.

The efficacy of blood transfusion depends on multiple RBC- and patient-related factors. Understanding the response to transfusion during sepsis is a challenging task. Targeting predetermined hemoglobin levels and/or macrohemodynamics is clearly not sufficient. Monitoring the microcirculation may get us closer to the answer [8].

## Abbreviations

HLA: Human leukocyte antigen
LD: Leukodepleted
nLD: Non-leukodepleted
RBC: Red blood cell
StO_2_: Tissue oxygen saturation
